# Ecological characteristics of the truncal skin mycobiome in acne and its association with doxycycline exposure

**DOI:** 10.71150/jm.2511019

**Published:** 2026-01-31

**Authors:** Hyun Ji Lee, Yong-Joon Cho, Nayan Jin, Piyapat Rintarhat, Won Hee Jung, Hei Sung Kim

**Affiliations:** 1 Department of Dermatology, Yeouido St. Mary's Hospital, The Catholic University of Korea, Seoul 06591, Republic of Korea; 2 Department of Molecular Bioscience, Kangwon National University, Chuncheon 24341, Republic of Korea; 3 Multidimensional Genomics Research Center, Kangwon National University, Chuncheon 24341, Republic of Korea; 4 Department of Dermatology, Incheon St. Mary's Hospital, The Catholic University of Korea, Seoul 06591, Republic of Korea; 5 Department of Systems Biotechnology, Chung-Ang University, Anseong 17546, Republic of Korea

**Keywords:** truncal acne, skin mycobiome, ITS2

## Abstract

Truncal acne significantly impairs quality of life yet remains underexplored relative to facial acne, particularly with respect to fungal ecology. The trunk represents a distinct cutaneous niche characterized by thicker epidermis, larger follicular units, and frequent occlusion, and harbors a high abundance of *Malassezia* species. In this study, we used internal transcribed spacer 2 (ITS2) amplicon sequencing to characterize the truncal mycobiome in patients with acne and in healthy controls and to compare fungal community features across doxycycline exposure groups. Although serial sampling was planned, seven participants contributed a single follow-up sample after doxycycline treatment, and only two participants contributed multiple follow-up samples sufficient for true within-subject longitudinal analyses; therefore, most analyses represent exposure-stratified cross-sectional comparisons rather than confirmed temporal change. At baseline, truncal acne lesions exhibited increased fungal richness and distinct community composition compared with controls. Acne lesions were more frequently enriched for *Malassezia globosa*, whereas healthy controls were dominated by *M. sympodialis*. Across doxycycline exposure groups, fungal communities remained *Malassezia*-dominant with substantial inter-individual variability. Doxycycline exposure was associated with partial and heterogeneous differences in *Malassezia* species composition without uniform normalization toward control profiles. Because only fungal sequencing was performed, bacterial–fungal interactions were inferred from prior literature and not directly measured. These findings indicate that truncal acne is associated with a distinct fungal community structure and highlight the need for integrated, longitudinal multi-omics studies to clarify treatment-associated microbial dynamics.

## Introduction

Truncal acne, affecting the chest, shoulders, and back, affects a substantial proportion of patients with acne vulgaris and can significantly impair quality of life (Ballanger et al., 2023; Menteşoğlu et al., 2025). Despite its prevalence, mechanistic studies of acne have predominantly focused on facial skin, whereas the trunk represents a biologically distinct environment with unique structural and ecological characteristics. Truncal skin differs from facial skin in follicular size, epidermal thickness, and exposure to occlusion from clothing, all of which influence microbial colonization and community structure (Aly et al., 1978; Otberg et al., 2004; Sandby-Møller et al., 2003).

The cutaneous mycobiome, dominated by lipid-dependent *Malassezia* species in sebaceous regions, has emerged as a potential contributor to inflammatory skin disease (Ayers et al., 2005; Findley et al., 2013; Martínez-Ortega et al., 2024; Park et al., 2021). Different *Malassezia* species exhibit distinct lipid utilization pathways, enzymatic activity, and immunomodulatory properties, suggesting that species-level composition may influence disease states (Li et al., 2022). While *M. sympodialis* commonly predominates on healthy truncal skin, *M. globosa* has been reported more frequently in acne-prone areas; however, data on fungal community structure in truncal acne remain limited (Grice and Segre, 2011; Prohic et al., 2016).

Systemic tetracyclines such as doxycycline are widely prescribed for moderate-to-severe truncal acne because of their antibacterial and anti-inflammatory properties (Woo and Kim, 2022). Although doxycycline lacks direct clinical antifungal activity, antibiotic exposure may indirectly influence fungal communities by altering the cutaneous environment or microbial competition. Most microbiome studies of acne treatment have focused on bacterial communities, and the effects of systemic antibiotics on the truncal mycobiome remain poorly defined.

In this study, we characterized the truncal fungal microbiome in patients with acne and healthy controls using ITS2 amplicon sequencing. We further compared fungal community features across groups stratified by doxycycline exposure. Because longitudinal sampling was limited, our analyses primarily reflect cross-sectional, exposure-stratified comparisons rather than within-subject temporal trajectories.

## Materials and Methods

### Study population

Patients with truncal acne were recruited from Incheon St. Mary’s Hospital, The Catholic University of Korea. Eligible participants had moderate-to-severe truncal acne (Investigator’s Global Assessment [IGA] grades 3–4). Exclusion criteria included systemic or topical antibiotic use within three months, prior isotretinoin therapy, pregnancy, and other inflammatory skin diseases. Healthy controls without acne or inflammatory dermatoses were also recruited. The study was approved by the institutional review board (IRB No. OC21TISI0158), and all participants provided written informed consent.

### Antibiotic treatment and exposure classification

Patients received oral doxycycline at a dose of 100 mg twice daily according to standard clinical practice for truncal acne, which typically requires prolonged systemic therapy extending beyond four months. For analytic purposes, a treatment “course” was defined as approximately six weeks of continuous therapy. Samples were stratified by doxycycline exposure history at the time of sampling (untreated, single course, or multiple courses). This stratification reflects cumulative treatment exposure rather than longitudinal tracking of the same individuals across defined treatment stages.

### Sample collection

Sampling was performed at four visits (V0–V3) at approximately 4–6-week intervals. At each visit, one or two fungal swabs were collected from the upper back using sterile cotton swabs (EASY SWAB, Hanil-Komed Inc., Korea) over a standardized 4 cm^2^ area with 20 strokes (10 horizontal, 10 perpendicular). All samples were collected by a single investigator (H.S.K.). Samples were obtained at baseline before antibiotic exposure and, in a subset of participants, at follow-up visits; seven participants provided a single post-treatment sample, and only two provided multiple follow-up samples sufficient for within-subject comparison.

### DNA extraction from skin swabs

Swabs were vortexed for 3 min, and 500 µl of each suspension was centrifuged at 12,000 rpm for 15 min at 4°C. The pellet was processed using the DNeasy PowerSoil Pro Kit (Qiagen, USA) (Rintarhat et al., 2024).

### Mycobiome amplicon sequencing and analysis

ITS2 libraries were prepared using ITS3/ITS4 primers, followed by Illumina adapter addition (Bellemain et al., 2010). Sequencing was performed with 250-bp paired-end reads on a MiSeq platform. Reads were trimmed using *Trimmomatic v0.36* (Bolger et al., 2014), merged with *PEAR v0.9.6* (Zhang et al., 2014), and analyzed in QIIME2 (Bolyen et al., 2019). Taxonomic classification was assigned against the UNITE *v10.0* database (Abarenkov et al., 2024). Statistical analyses and visualization were conducted in R using phyloseq (McMurdie and Holmes, 2013) and MicrobiomeAnalyst (Lu et al., 2023).

### Statistical analysis

Alpha diversity was assessed using Chao1 richness and Shannon diversity indices. Beta diversity was evaluated using Bray–Curtis dissimilarity and principal coordinates analysis. Group comparisons were performed using non-parametric tests with false discovery rate correction where appropriate. Baseline acne severity (IGA grade), age, and sex were recorded but were not included as covariates in statistical models because of limited sample size and the absence of longitudinal transitions within individuals.

## Results

### Study population and demographics

A total of 37 baseline samples were analyzed, including 27 samples from 19 patients with truncal acne and 10 samples from healthy controls. The mean age was 28.4 years in the acne group (32% female) and 36.6 years in the control group (90% female) (Table S1). These demographic differences were not controlled for in statistical analyses and may influence microbial composition.

### Fungal community composition

**Fig. 1.**
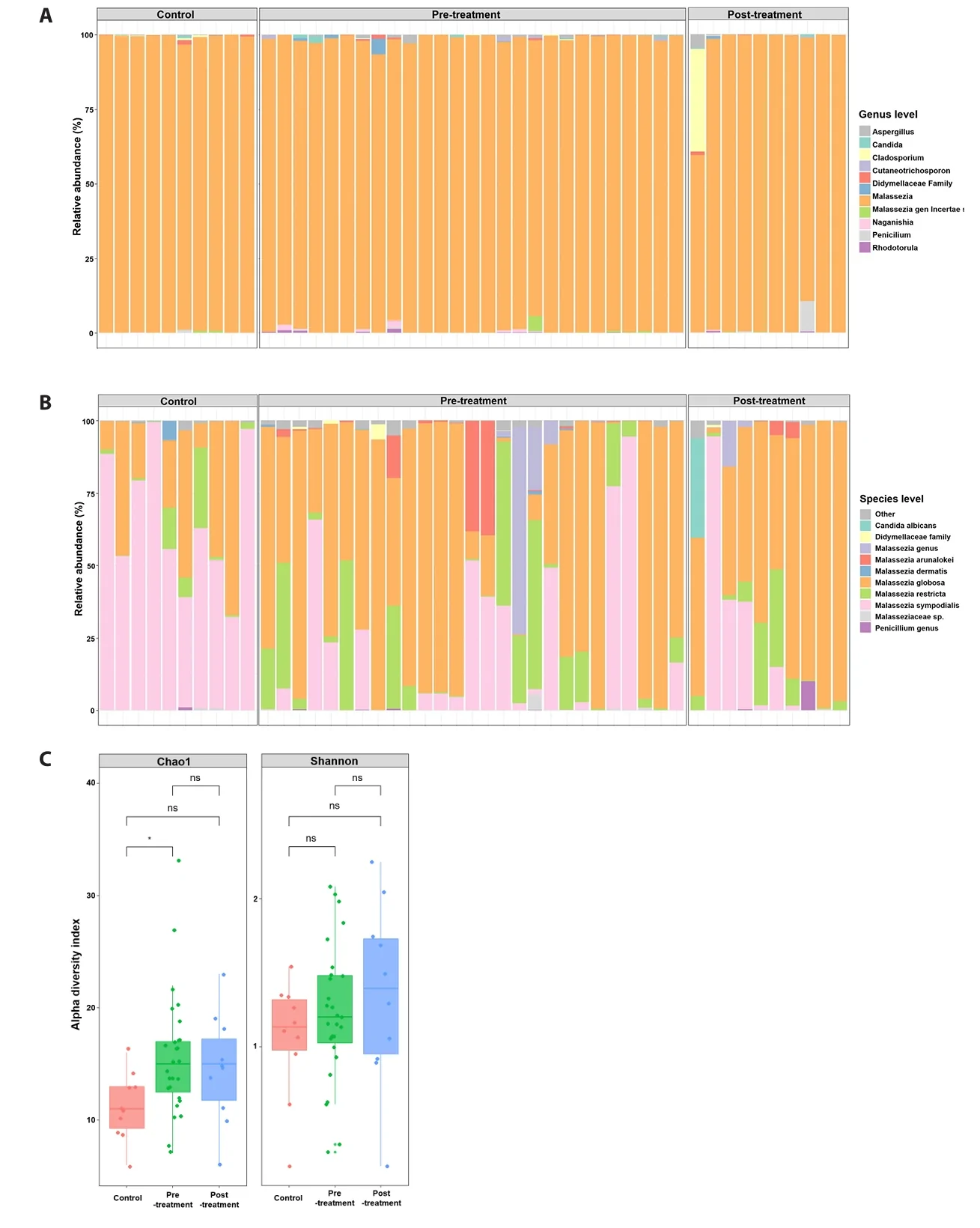
Community composition and α-diversity. Relative abundance of fungal taxa and alpha diversity metrics across untreated acne, doxycycline-exposed acne samples, and healthy controls. Richness differs between groups, whereas Shannon diversity does not show significant differences.

ITS2 sequencing identified 725 unique fungal taxa across all samples. Figure 1 summarizes baseline (pre-doxycycline exposure) and post-doxycycline exposure samples from acne patients alongside healthy controls. At the genus level (Fig. 1A), fungal communities in all groups were dominated by *Malassezia*, although acne samples exhibited greater taxonomic heterogeneity and occasional non-*Malassezia* reads. At the species level (Fig. 1B), control skin was largely dominated by *M. sympodialis*, with minor contributions from *M. globosa*. In contrast, baseline acne lesions displayed more variable species compositions, including *M. globosa*, *M. sympodialis*, *M. restricta*, *M. arunalokei*, and unclassified *Malassezia* taxa.

### Alpha and beta diversity

**Fig. 2.**
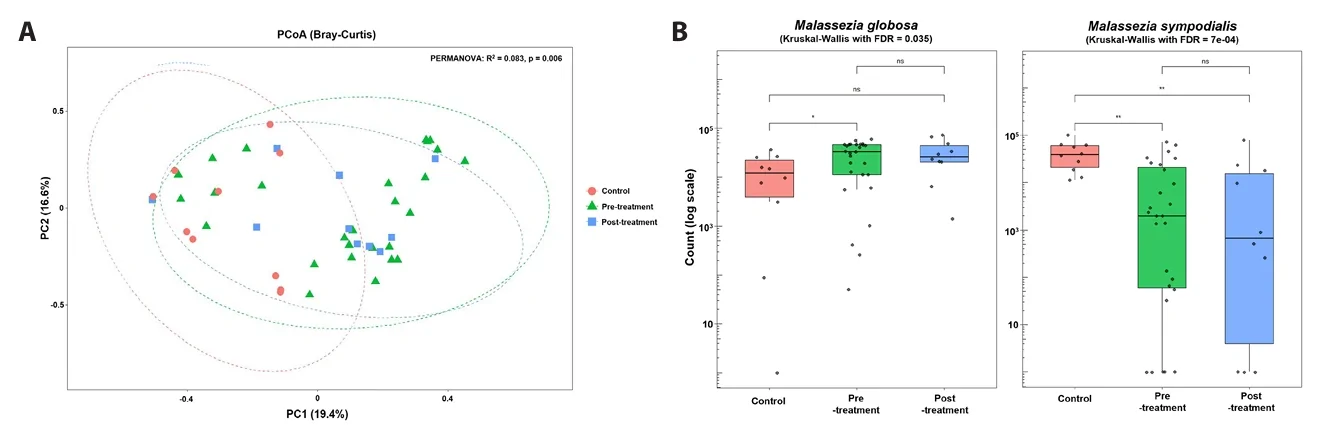
Beta diversity and dominant species. Principal coordinates analysis of Bray–Curtis dissimilarity comparing fungal community composition among untreated acne, doxycycline-exposed acne samples, and controls. Group separation reflects cross-sectional differences rather than within-subject temporal change.

Alpha-diversity analysis (Fig. 1C) demonstrated significantly higher Chao1 richness in baseline acne samples compared with controls (*p* < 0.05), whereas Shannon diversity did not differ significantly between groups. Principal coordinates analysis of Bray–Curtis dissimilarities (Fig. 2A) revealed overall separation between acne and control samples (PERMANOVA, *p* = 0.006), indicating distinct fungal community structures. Analysis of dominant taxa (Fig. 2B) showed significantly higher abundance of *M. globosa* and lower abundance of *M. sympodialis* in baseline acne samples compared with controls (Kruskal–Wallis test with FDR correction: *M. globosa* FDR = 0.035; *M. sympodialis* FDR = 7 × 10^-4^).

### Comparison across doxycycline exposure groups

When stratified by doxycycline exposure, fungal communities remained *Malassezia*-dominant and exhibited substantial inter-individual variability. Relative abundance of dominant *Malassezia* species differed between untreated acne samples and samples obtained after doxycycline exposure; however, post-exposure profiles did not consistently resemble those of healthy controls (Fig. 1A and 1B). Post-exposure samples remained heterogeneous, and although several samples were positioned closer to the control centroid in ordination space, complete overlap was not observed. Chao1 richness in post-exposure samples was numerically higher and more variable than in controls; however, neither the post-exposure versus control comparison nor the direct pre-versus post-exposure comparison reached statistical significance (Fig. 1C).

### Exposure-stratified patterns

**Fig. 3.**
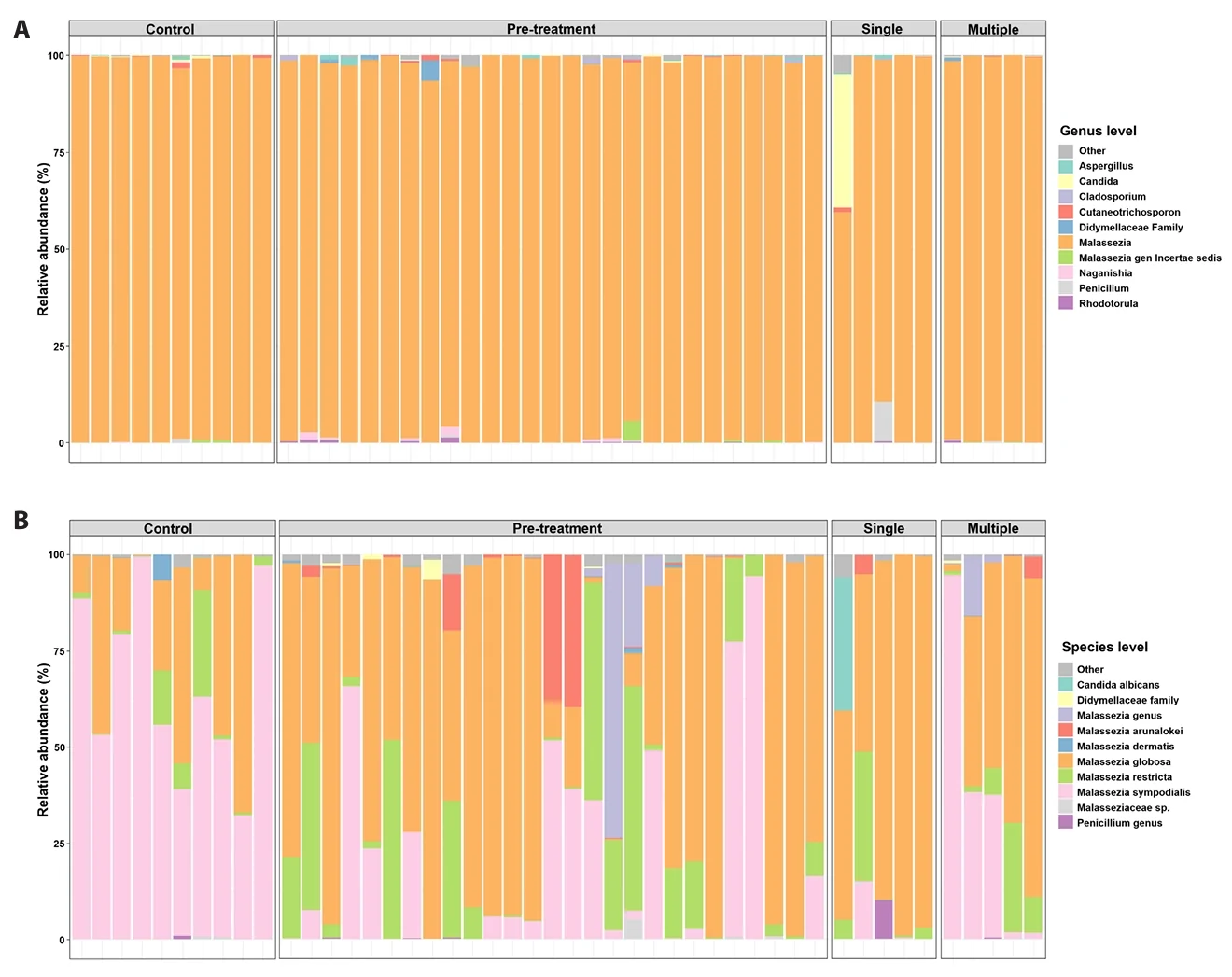
Course-stratified species composition (single vs multiple courses). Species-level *Malassezia* composition stratified by doxycycline exposure history (single vs multiple courses) and controls. These exposure-stratified comparisons are descriptive and do not represent longitudinal recovery trajectories.

**Fig. 4.**
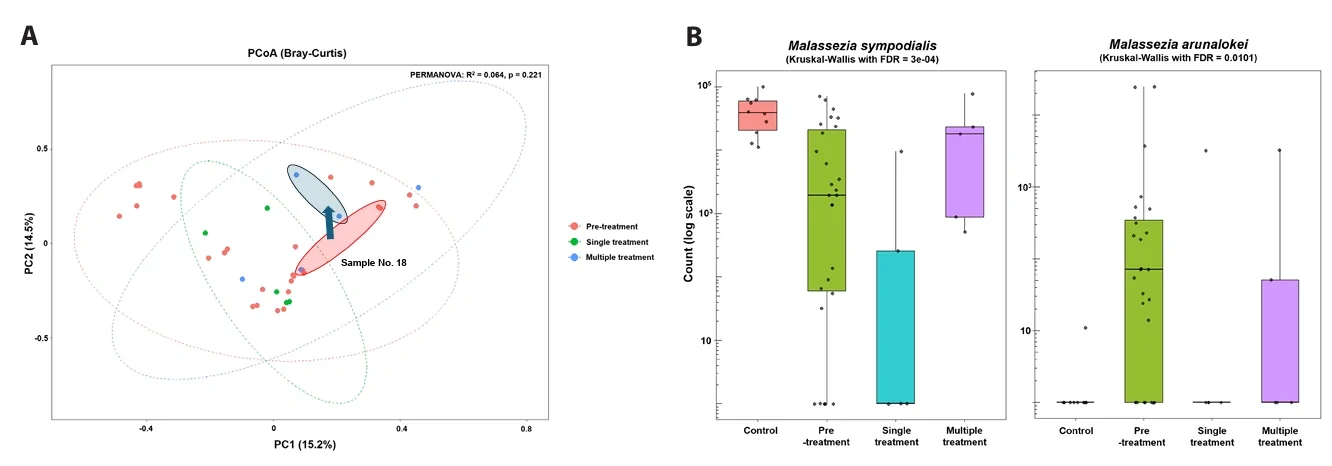
Course-stratified community dynamics and species trajectories. Beta diversity and relative abundance patterns across exposure groups. Observed differences may reflect baseline heterogeneity or treatment-selection bias and should not be interpreted as causal treatment effects.

To further examine exposure-associated differences, post-exposure acne samples were stratified by doxycycline course number (single versus multiple) (Fig. 3A and 3B). Comparisons between single- and multiple-course exposure groups reflect descriptive, exposure-stratified cross-sectional patterns and should not be interpreted as evidence of treatment-induced recovery or confirmed temporal change (Figs. 3 and 4). Bray–Curtis PCoA did not demonstrate statistically significant global separation by exposure group (PERMANOVA, *p* = 0.221) (Fig. 4A). At the species level, samples from individuals exposed to a single course generally remained *M. globosa*–biased with relative depletion of *M. sympodialis* compared with controls, whereas samples from individuals exposed to multiple courses showed higher relative abundance of *M. sympodialis* and lower abundance of *M. arunalokei* (Fig. 3B). Across exposure groups, differences in *M. sympodialis* and *M. arunalokei* reached statistical significance; however, these findings should be interpreted as exposure-associated differences rather than evidence of longitudinal normalization. Collectively, these results indicate heterogeneous fungal community patterns associated with doxycycline exposure, without uniform convergence toward a control-like state (Fig. 4B).

## Discussion

This study demonstrates that truncal acne is associated with a distinct fungal community structure compared with healthy truncal skin, characterized by greater heterogeneity and altered *Malassezia* species composition. Acne lesions exhibited higher fungal richness and more variable species profiles, with frequent enrichment of *M. globosa* and relative depletion of *M. sympodialis*, whereas healthy control skin was consistently dominated by *M. sympodialis*. Together, these findings support the presence of fungal community differences in truncal acne, although the cross-sectional nature of the data limits inference regarding causality or temporal progression.

Regional skin biology likely contributes to these observed differences. The trunk differs from facial skin in epidermal thickness, follicular architecture, and exposure to occlusion from clothing, resulting in a relatively stable, lipid-rich microenvironment that favors lipid-dependent fungi (Aly et al., 1978; Otberg et al., 2004; Tagami, 2008). Prior surveys have consistently reported a high abundance of *Malassezia* species on the torso, suggesting that this anatomical niche may accentuate species-level ecological selection (Akaza et al., 2010; Faergemann et al., 1983; Findley et al., 2013). Our findings extend these observations by demonstrating that truncal acne is associated not only with *Malassezia* dominance but also with greater compositional variability at the species level.

Within this niche, the relative abundance of *M. globosa* in acne lesions is consistent with known biological differences among *Malassezia* species. Experimental studies indicate that *M. globosa* can activate pro-inflammatory pathways, including NLRP3 inflammasome signaling and IL-1β production in sebocytes and keratinocytes, whereas *M. sympodialis* appears to induce a more chemokine-skewed host response (Li et al., 2022; Park et al., 2021; Wolf et al., 2021). In addition, *M. globosa* exhibits broader lipase repertoires and higher extracellular lipase activity than *M. sympodialis*, which may influence lipid utilization in sebaceous environments (DeAngelis et al., 2007; Juntachai et al., 2009; Wu et al., 2015). Although our study did not assess host responses or functional activity, these species-specific properties provide a plausible framework for understanding why *M. globosa* may be more frequently associated with acne lesions under occlusive, lipid-rich conditions. Direct evidence linking these mechanisms to truncal acne in vivo, however, remains limited.

Across doxycycline exposure groups, fungal communities remained *Malassezia*-dominant with substantial inter-individual variability. Differences in species composition were observed between untreated acne samples and samples obtained after doxycycline exposure, but post-treatment profiles did not consistently resemble those of healthy controls. Richness remained variable following treatment, and neither the post-treatment versus control comparison nor the direct pre- versus post-treatment comparison reached statistical significance.

When post-treatment samples were stratified by doxycycline course number, differences in *Malassezia* species composition were observed between single- and multiple-course exposure groups. Importantly, because only two participants contributed sufficient repeated samples for within-subject analysis, these findings primarily reflect exposure-stratified cross-sectional differences rather than confirmed temporal change; thus, any apparent trends should be interpreted cautiously. Comparisons between single- and multiple-course exposure groups therefore represent descriptive, exposure-associated patterns and should not be interpreted as evidence of treatment-induced recovery or longitudinal normalization. Although samples from individuals with multiple courses showed higher relative abundance of *M. sympodialis* and lower abundance of *M. arunalokei*, global community separation by exposure group was not statistically significant, underscoring the heterogeneous and incomplete nature of these differences.

Potential mechanisms linking antibiotic exposure to fungal community structure remain speculative. This study employed ITS2 sequencing exclusively and did not directly measure bacterial abundance, sebum composition, or other environmental factors. Consequently, bacterial–fungal interactions were inferred from prior literature rather than assessed directly. Previous studies, including our own work in facial acne, have shown that doxycycline can reduce *Cutibacterium acnes* abundance and alter bacterial community structure; however, those findings were derived from a distinct anatomical site and cohort and cannot be extrapolated to truncal skin. Integrated bacteriome–mycobiome–lipidomic studies will be necessary to determine whether indirect ecological effects contribute to the fungal patterns observed here.

Several limitations should be acknowledged. The longitudinal subset was small, limiting the ability to assess within-subject dynamics. Demographic differences between acne and control groups were not controlled for and may influence microbial composition. Sequencing was restricted to the fungal community, precluding analysis of cross-kingdom interactions, and ITS2 profiling does not provide strain-level or functional resolution. Sebum quantity and composition were not measured, and concomitant topical therapies may have influenced microbial communities. Although fungal folliculitis was considered, clinical improvement with doxycycline—an antibiotic without direct clinical antifungal activity—supports truncal acne as the predominant diagnosis.

In summary, truncal acne is associated with distinct fungal community features characterized by increased richness and altered *Malassezia* species composition. Doxycycline exposure is associated with heterogeneous, exposure-stratified differences in the truncal mycobiome but does not result in uniform convergence toward a control-like state. These findings highlight the unique ecological context of truncal skin and underscore the need for future longitudinal, multi-omics studies to clarify the role of fungal communities in truncal acne and to inform ecology-aware therapeutic strategies.

## Supplementary Information

Table S1. Demographic data on the truncal acne and control population
