# Supplementary material for: Ecological characteristics of the truncal skin mycobiome in acne and its association with doxycycline exposure
Source: J Microbiol. 2026 Jan 31;64(1):e2511019. doi: 10.71150/jm.2511019 (PMC13577103; doi:10.71150/jm.2511019)
Supplement: Supplementary file 1 [file jm-2511019-Supplementary-Table-S1.pdf]

Table S1. Demographic data on the truncal acne and control population

Truncal acne patients

No.	Sample No.	Sex	Age	V0	V1	V2	V3
1	1	F	26	O			
2	4	M	20	O			
3	7	M	22	O			
4	9	M	20	O			
5	10	F	17	O			
6	12	M	29	O			
7	13	F	33	O			
8	14	M	21	O			
9	15	M	25	O			
10	16	M	45	O			
11	17	F	48	O	O	O	
12	18	F	16	O		O	
13	19	F	18	O		O	O
14	20	M	17	O			
15	21	M	53	O			
16	22	M	34	O	O	O	
17	23	F	30	O	O		
18	24	M	27	O	O		
19	25	M	43	O	O		

Control population

No.	Sex	Age
1	M	31
2	F	31
3	F	27
4	F	29
5	F	48
6	F	49
7	F	28
8	F	43
9	F	35
10	F	45
